# Supplementary material for: Characterization of the Hydrolysis Kinetics of Fucosylated Glycosaminoglycan in Mild Acid and Structures of the Resulting Oligosaccharides
Source: Mar Drugs. 2020 May 29;18(6):286. doi: 10.3390/md18060286 (PMC7345840; doi:10.3390/md18060286)
Supplement: Supplementary file 1 [file marinedrugs-18-00286-s001.pdf]

Supplementary data

Characterization of the Hydrolysis Kinetics of Fucosylated Glycosaminoglycan in Mild Acid and Structures of the Resulting Oligosaccharides

Xixi Liu [1], Zhexian Zhang [1], Hui Mao [2], Pin Wang [1], Zhichuang Zuo [1], Li Gao [1], Xiang Shi [1], Ronghua Yin [2], Na Gao [1] and Jinhua Zhao [1,2,*]

[1] School of Pharmaceutical Sciences, South-Central University for Nationalities, Wuhan 430074, China; Lxx201709@163.com (X. L.); Nataliezz@163.com (Z. Z); wangpin1994@163.com (P. W.); 18271682301@163.com (Z. Z.); glp1284813702@163.com (L. G.); xiangshi041@gmail.com (X. S.).

[2] State Key Laboratory of Phytochemistry and Plant Resources in West China, Kunming Institute of Botany, Chinese Academy of Sciences, Kunming 650201, China; maohui@mail.kib.ac.cn (H.M.); yinronghua@mail.kib.ac.cn (R.Y.)

* Correspondence: gn2008.happy@163.com (N.G.); zhao.jinhua@mail.kib.ac.cn (J.Z).

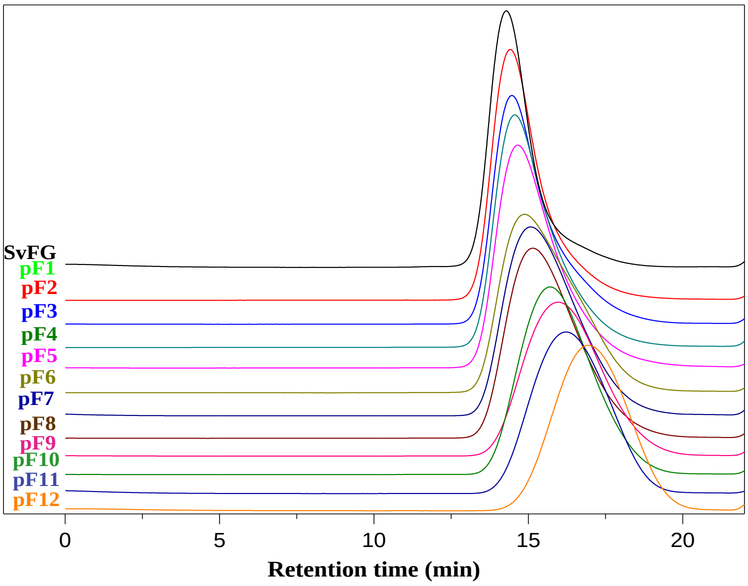

Figure S1. The HPGPC profiles of native SvFG and pF1-12. The pF1-12 was the partial defucosylated derivatives from SvFG which was hydrolyzed in 0.1 M H_2SO_4 at 60 °C.

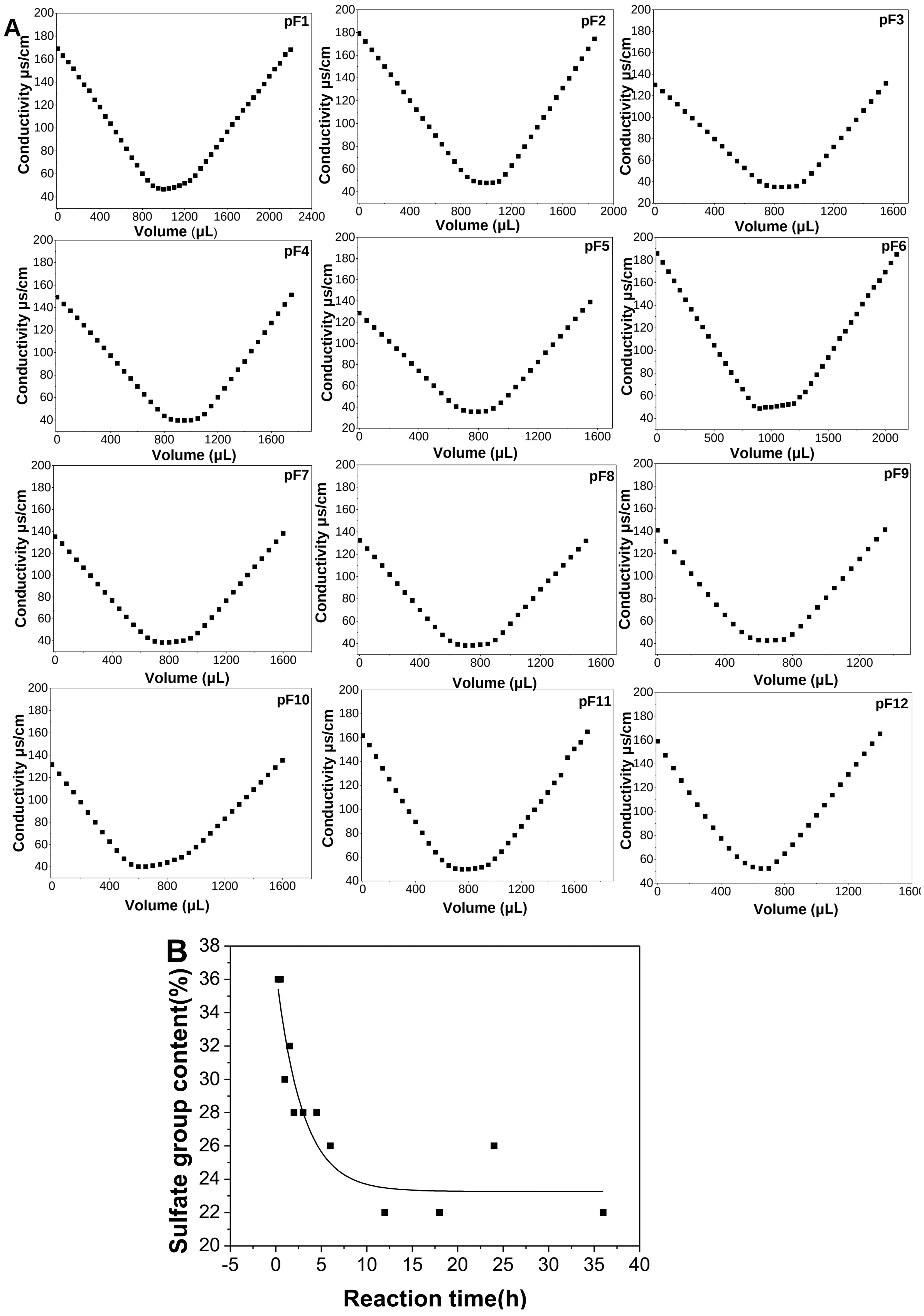

Figure S2. Conductimetric titration curves of pF1-12 (A); the fitting curve of SO_3^- content of pF1-12 with reaction time (B).

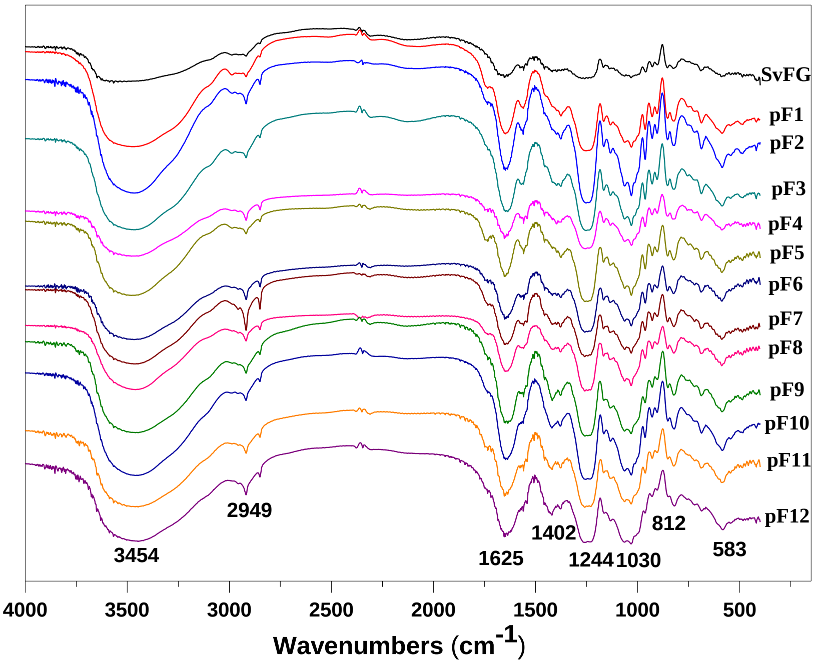

Figure S3. The IR spectra of native SvFG and pF1-12

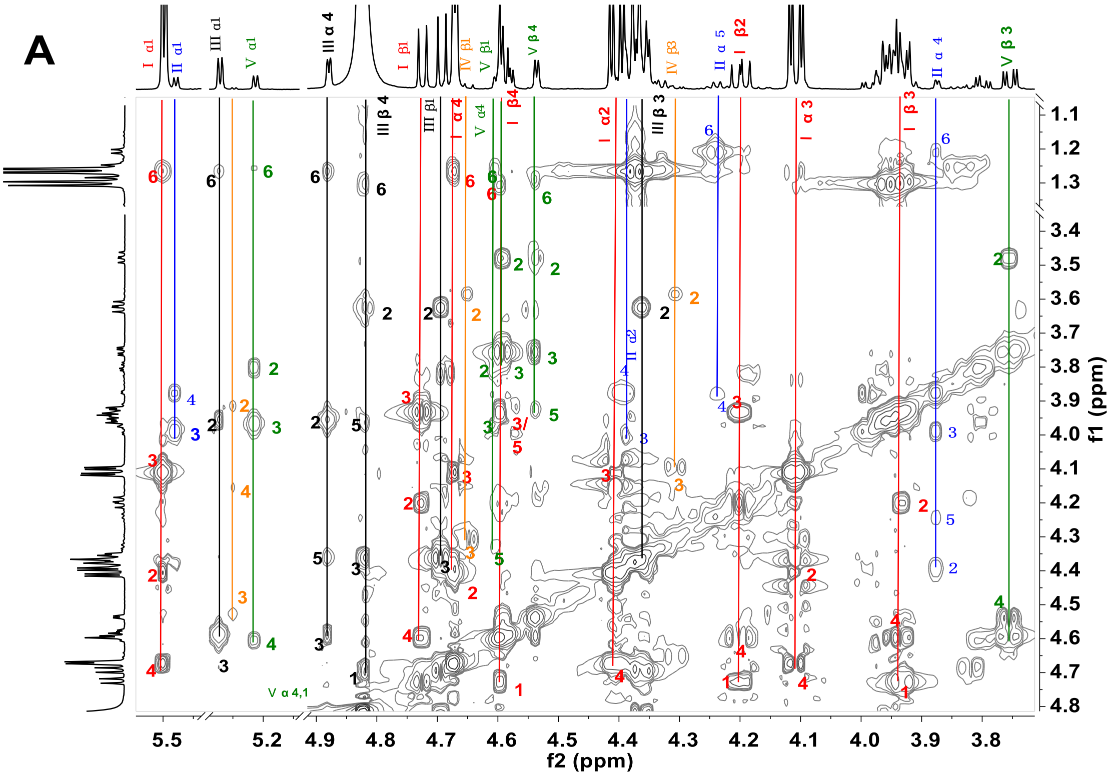

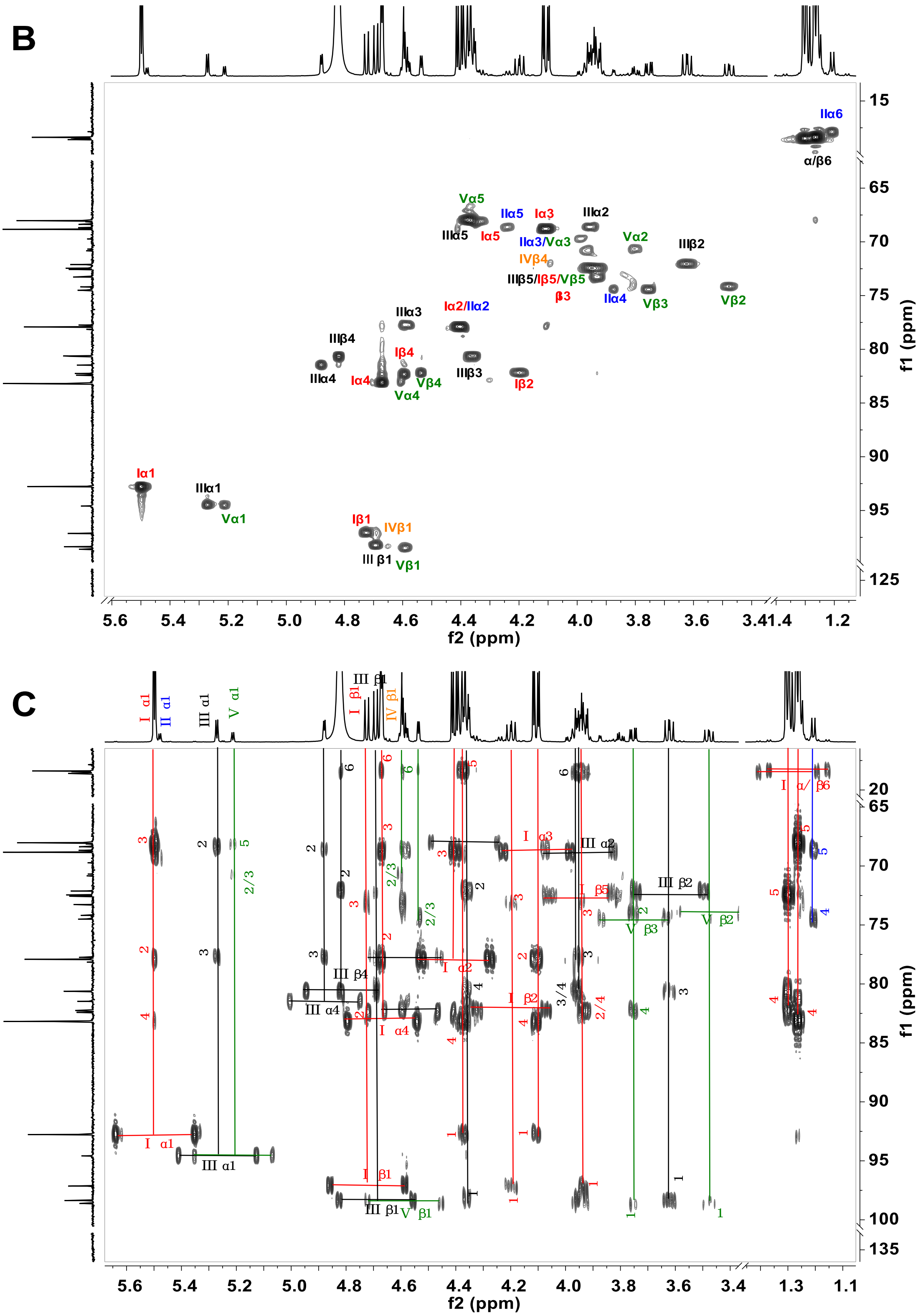

Figure S4. The ^{1}H-^{1}H TOCSY (A), ^{1}H-^{13}C HSQC (B) and HMBC (C) spectra of Sc8

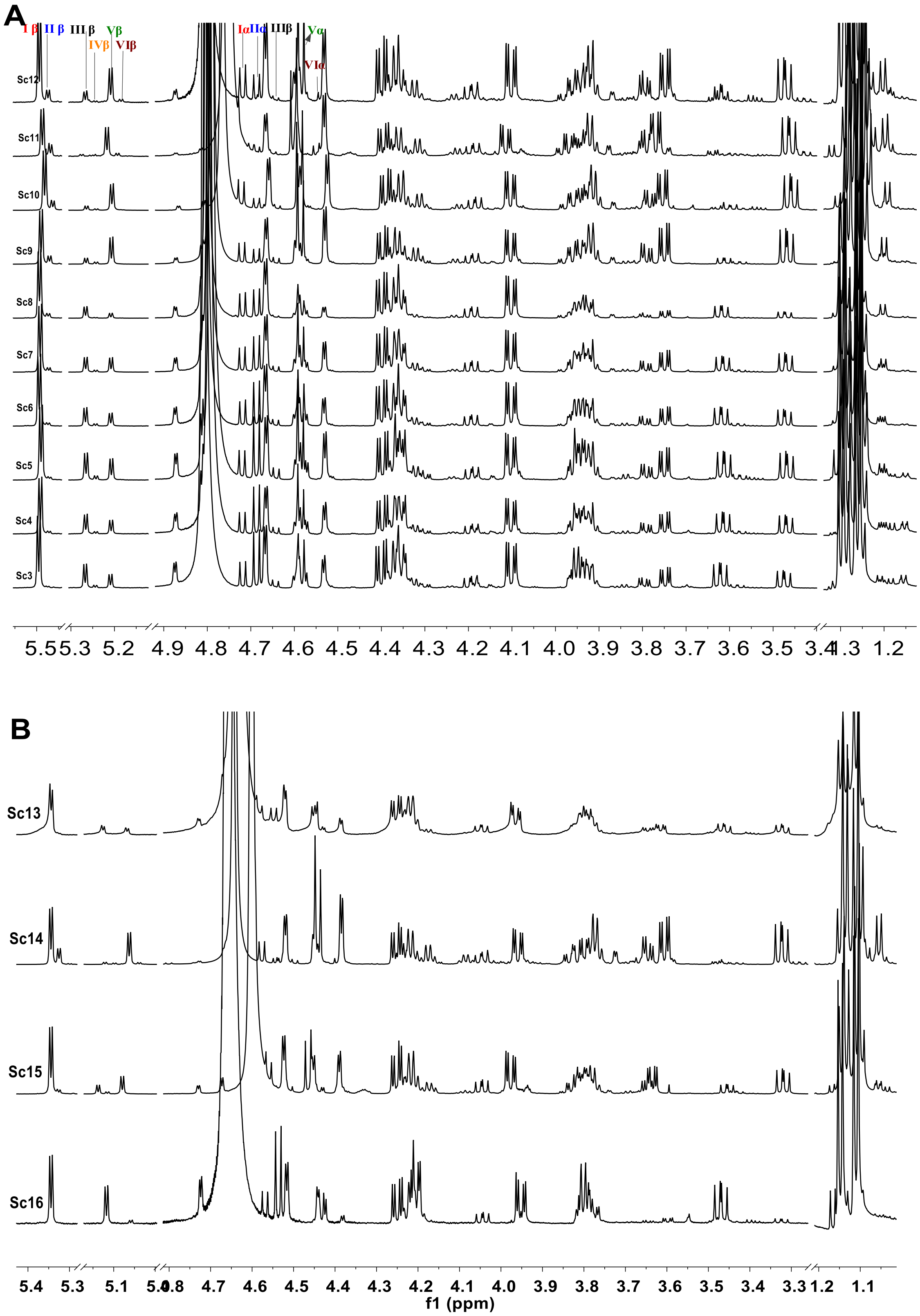

Figure S5. The ^{1}H NMR spectra of Sc3-12 (A) and Sc13-16 (B)

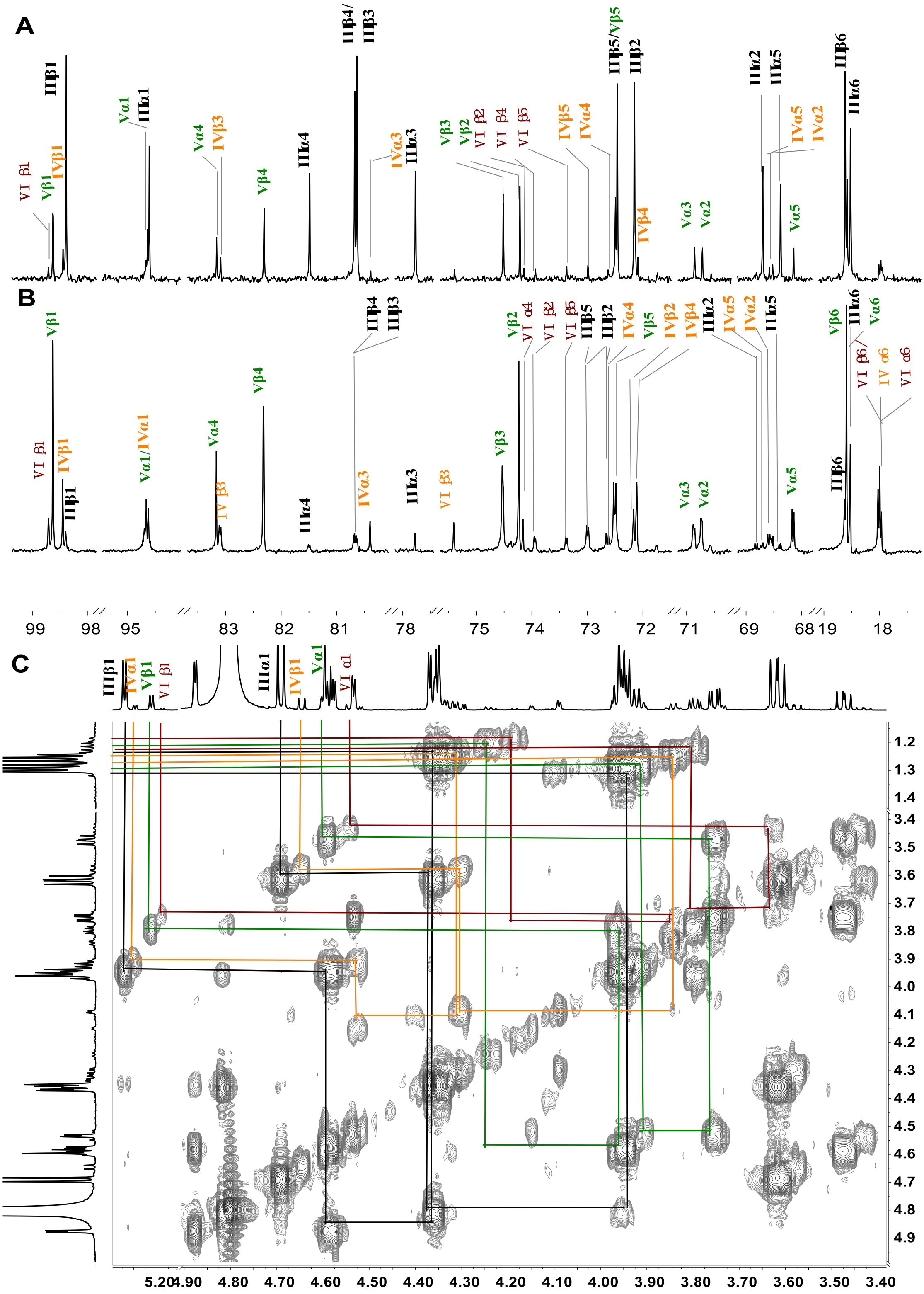
A
B
C
IIIβ1
IIIα1
IIIβ4/
IIIβ3
IIIβ5/Vβ5
IIIβ2
IIIα2
IIIα5
IIIβ6
IIIα6
Vβ1
Vα1
Vα4
Vβ4
IVβ1
IVβ3
IVα3
IVα4
IVβ5
IVβ4
IVα1
IVα5
IVα2
Vα3
Vα2
Vα5
Vβ3
Vβ2
Vβ6
Vα6
VI β1
VI α1
VI β2
VI β4
VI β5
VI β6
VI α6
IV α6
99
98
95
83
82
81
78
75
74
73
72
71
69
68
19
18
5.20
4.90
4.80
4.70
4.60
4.50
4.40
4.30
4.20
4.10
4.00
3.90
3.80
3.70
3.60
3.50
3.40
1.2
1.3
1.4

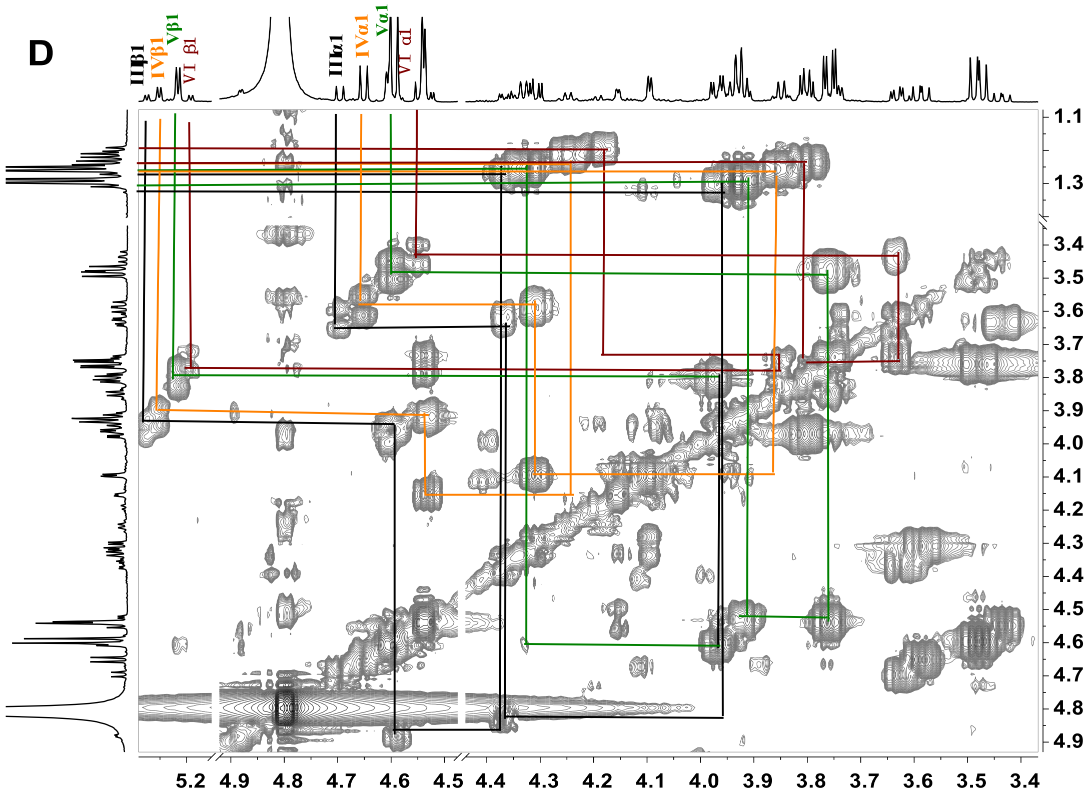

Figure S6. The ^{13}C NMR, ^{1}H-^{1}H COSY spectra of Sc19 (A, C) and Sc20 (B, D). Sc19 and Sc20 were the acid released fucose branches of HfFG at 60 °C for 12 h and 100 °C for 2 h, respectively. Lables I, II, III, IV, V and VI represent type I (Fuc2S4S), II (Fuc2S), III (Fuc3S4S), IV (Fuc3S), V (Fuc4S) and VI (Fuc) of FucS, respectively.
